# The value of dynamic changes in FT3 level for predicting 90-day prognosis of HBV-ACLF patients

**DOI:** 10.1186/s40001-024-01770-2

**Published:** 2024-05-15

**Authors:** Jian Zhang, Luxue Zhang, Xiaokang Fu, Yu Chen, Zhongping Duan, Geng Tian

**Affiliations:** 1 Infectious Diseases Department, Capital Medical University XuanWu Hospital, No. 45 of Changchun Street, Xicheng District, Beijing, 100053 China; 2 Department of Difficult and Complicated Liver Diseases and Artificial Liver Center, Beijing Youan Hospital, Capital Medical University, Beijing, 100069 China

**Keywords:** Acute-on-chronic liver failure, FT3, Dynamic changes, Prognosis

## Abstract

**Objective:**

To explore the effect of dynamic changes in free triiodothyronine (FT3) level for predicting the 90 day prognosis of patients with hepatitis B virus–related acute-on-chronic liver failure (HBV-ACLF).

**Methods:**

The clinical data of 122 hospitalised patients with HBV-ACLF between September 2018 and January 2020 were collected and divided into a survival group (77 cases) and a death group (45 cases) according to the 90 day prognosis. We statistically analysed the characteristics of FT3 changes in the two groups of patients. Binary logistic regression one-way analysis was used to assess the degree of influence of each factor. The Kaplan–Meier survival curve and receiver operating characteristic curve were used to evaluate the effect of a single change in FT3 level difference (single △FT3) and the FT3 level change range (△FT3 range) in predicting the 90-day prognosis of patients.

**Results:**

There were only three types of changes in FT3 levels, which included 19 (15.6%) cases of continuous normal type, 35 (28.7%) cases of continuous decrease type and 68 (55.7%) cases of U-shaped change type. The difference in survival curves between the three types of patients was statistically significant (*P* < 0.001).

**Conclusion:**

The dynamic change type of FT3 is related to the disease severity and 90-day prognosis of patients with HBV-ACLF. The single FT3 value and FT3 range could be used as a predictive factor for the 90-day prognosis of patients with HBV-ACLF. These results have a degree of research value and are worth further exploration in the future.

## Introduction

Acute-on-chronic liver failure (ACLF) is a clinical syndrome of liver failure caused by acute injury due to chronic liver disease, which may be accompanied by multiple organ failure, posing a major threat to public health [1]. To date, disputes remain between the East and the West on the aetiology of chronic liver disease, the causes of liver disease and the inducement of acute injury, which makes it difficult to unify a definition and diagnostic criteria of ACLF. The Chinese Society of Hepatology (CSH) guidelines define ACLF based on specific criteria related to jaundice, coagulation and organ failure, reflecting the unique clinical characteristics observed in the Chinese population. The American Association for the Study of Liver Diseases guidelines may include differences in its criteria for an ACLF diagnosis. The European Association for the Study of the Liver guidelines provide a European perspective on ACLF diagnosis. In this study, Potential variations in criteria are considered and comparisons will be made to understand regional differences. Given the focus on the Asia–Pacific region of the current researches, the Asian Pacific Association for the Study of the Liver (APASL) guidelines may evidence distinct criteria. Accordingly, we selected the diagnostic criteria presented in the CSH guidelines. The study considers these differences to assess the generalisability of the findings to a broader patient population. Scholars recognise that the mortality rate of this disease is high [2], exceeding 50% [3]. Hepatitis B virus–related ACLF is the most important type of ACLF in China [4], accounting for approximately 80% of ACLF, with a high fatality rate of 60–80% [5, 6]. For the treatment of ACLF, except for liver transplantation, there is still a lack of internationally recognised specific drugs and methods [7]. Clinical studies have shown that about half of ACLF is potentially reversible, and some patients can achieve a full recovery of the disease through comprehensive medical treatment; moreover, organ function can be recovered from an acute shock to the pre-disease state, without the need for a liver transplantation [8, 9]. Therefore, establishing an effective analytical model for objectively predicting the prognosis of patients with HBV-ACLF and accurately reflecting the patient's condition, identifying differences in the prognosis and selecting an individualised diagnosis and treatment plan suitable for the condition are key issues in the treatment of HBV-ACLF.

Although a variety of scoring systems have been developed to assess the prognosis of ACLF, most of them are calculated based on clinical parameters at diagnosis or baseline measurements of patients on admission [10]. Due to the variability in disease course lengths before admission and the pronounced difference in treatment approaches, the detection value at the time of admission alone cannot reflect the initial disease state of patients. Continuous assessment at multiple time points can, theoretically, more accurately reflect the clinical course and response to drug therapy, with higher prognostic power than using a single time point [11]. Many studies have shown that the use of dynamic data can more accurately reflect changes in patients' conditions and, accordingly, predict their prognosis [12, 13].

There exists a robust biological foundation for the investigation of thyroid hormones, particularly free triiodothyronine (FT3), in liver-related diseases [14–17]. The liver plays a crucial role in thyroid hormone metabolism and regulation. In the context of liver dysfunction, alterations in thyroid hormone levels are plausible due to impaired synthesis, metabolism or clearance. Thyroid hormones, particularly thyroid stimulating hormone (TSH), influence various metabolic processes and disturbances in their levels may reflect the severity of liver dysfunction [18–20]. Although direct evidence on the association between FT3 and an HBV-ACLF prognosis may be lacking in the literature, previous studies have highlighted the clinical relevance of thyroid hormones in liver diseases [16, 18]. Perturbations in thyroid hormone levels have been observed in chronic liver diseases and their impact on patient outcomes is an area of growing interest. Recognising the potential role of FT3 as a predictor in the specific context of HBV-ACLF is an essential step in addressing critical gaps in our understanding of the disease.

This study selected patients with HBV-ACLF who were hospitalised between September 2018 and January 2020 in Beijing You'an Hospital, affiliated with Capital Medical University, to conduct an observational case-control study. This study aimed to investigate the predictive value of both individual FT3 values and the range of FT3 level changes (△FT3 range) concerning the 90  day prognosis of patients with HBV-ACLF. The research provides valuable insights into the prognostic potential of dynamic FT3 changes and offers a foundation for potential clinical applications in the management of patients with HBV-ACLF.

## Materials and methods

### Research participants

A total of 155 patients with HBV-ACLF who were hospitalised between September 2018 and January 2020 in Beijing You'an Hospital, affiliated with Capital Medical University, were selected. A total of 27 cases were excluded according to the exclusion criteria (2 cases were complicated with chronic HCV infection prior to a diagnosis of ACLF, 12 cases were complicated with drug-related, alcohol-related and metabolic liver diseases, 5 cases were complicated with liver cancer, 5 cases were complicated with heart failure and kidney injury and 3 cases were complicated with thyroid disease. The 90 day follow-up was not completed in 6 cases; 122 patients with HBV-ACLF were finally included. Inclusion criteria: (1) Age: 18–70 years old, no gender restrictions; (2) met the diagnostic criteria of HBV-ACLF, in this instance, HBV surface antigen-positive for more than 6 months, as well as patients who were still positive for HBV deoxyribonucleic acid on admission; (3) inclusion was limited to patients with a confirmed diagnosis of HBV-ACLF. Diagnosis criteria aligned with the CSH guidelines [21]. (4) Patients with comprehensive clinical data, including FT3 levels, were included. Exclusion criteria: (1) patients diagnosed with other hepatitis virus infections before a diagnosis of ACLF; (2) patients with drug-induced liver injury, alcoholic liver injury, autoimmune liver disease, inherited metabolic liver disease (such as Wilson’s disease and haemochromatosis); (3) patients with primary liver cancer or other malignant tumours; (4) patients who have used thyroxine-containing drugs or thyroxine-suppressing drugs within the preceding 3 months; (6) pregnant or lactating women. This study was approved by the Ethics Committee of our hospital (Ethics No. [2019]; Scientific Research Quick Review No. [989]. All patients provided signed informed consent and voluntarily participated in this study.

### Research methods

The patients included in the study were followed up for 90 days and were divided into a survival group (n = 77) and a death group (n = 45) according to prognosis. The survival group included transplant-free and transplant-survived patients. The death group included those who died during hospitalisation, those who stopped medical treatment and were discharged automatically due to disease progression, as well as those who had undergone liver transplants. General information about the patients was collected from 2 studies using the case management system of Beijing You'an Hospital, including gender, age, basis of liver disease (chronic hepatitis B/cirrhosis) and the cause of this liver failure (e.g. HBV reactivation, spontaneous bacterial peritonitis, alcohol consumption, oesophagogastric varices bleeding, acute upper respiratory tract infection, adult community-acquired pneumonia, drug-induced liver injury and acute kidney injury). After admission, the fasting FT3 level in the morning was detected every 4 days, and the Child–Turcotte–Pugh (CTP), model for end-stage liver disease (MELD), MELD-Na, Chronic Liver Failure Sequential Organ Failure Assessment (CLIF-SOFA), Chronic Liver Failure Organ Failure (CLIF-OF) and Acute-on-chronic Liver Failure Research Consortium (AARC) scores were recorded. Post-treatment follow-up was performed on admitted patients. The endpoint of follow-up was 90 days after the patient had been admitted to hospital. The main method of follow-up was by telephone according to the contact information provided at the patient’s registration. For patients who failed the telephone follow-up, their readmission records or outpatient follow-up records were rechecked. The 90 day survival status, time of death, date of liver transplantation and the number of days from admission to death/liver transplantation were recorded in detail.

### Laboratory tests

All blood samples were tested at the Clinical Inspection Center of Beijing You'an Hospital. Whole blood cell analysis was performed by flow cytometry using a Sysmex XE-2100 automatic blood cell analyser (Sysmex, Japan). Liver function testing was performed using a Japanese OTLCmpus AU5400 (Olympus, Tokyo, Japan) automatic biochemical analyser, including Alanine Aminotransferase (ALT), Aspartate Aminotransferase (AST), Total Bilirubin (TBil), albumin (ALB), Creatinine (Cr), Na and Lactic Acid (LA) tests. Coagulation function was detected using a Japan Sysmex CA-7000 automatic blood coagulation instrument, including prothrombin time (PT), prothrombin activity (PTA) and international normalized ratio (INR). For blood gas analysis, an M840 gas analyser was used. Five tests were conducted for hepatitis B as follows. The ELISA method was used to detect the markers using a kit provided by Kehua Company. For HBV-DNA detection, a fluorescence quantitative polymerase chain reaction (QT-PCR) was used using a kit provided by Daan Company and the 7500 QT-PCR instrument was used for detection. For FT3 detection, an Abbott Architect I2000SR automatic chemiluminescence analyser was used. The free triiodothyronine assay kit (chemiluminescence microparticle immunoassay) kit produced by Abbott Company was used, and the normal value reference range was 2.63–5.7 pmol/L.

### Treatment after admission

Antiviral therapy: According to the treatment recommendations provided in the Guidelines for the Prevention and Treatment of Chronic Hepatitis B (2019 Edition) [22], all patients received oral antiviral therapy with nucleoside (acid) analogues. In addition, conventional treatment was provided including liver protection, plasma infusion, ALB and nutritional support. According to the guidelines for diagnosis and treatment of related diseases, patients were given artificial liver plasma exchange therapy. Patients with hepatorenal syndrome or renal failure received ALB, terlipressin and/or renal replacement therapy. Patients with hepatic encephalopathy (HE) were treated with lactulose, ornithine aspartate, arginine and rifaximin. Bacterial infections were treated with antibiotics. Patients in shock received blood volume expansion, vasoactive drugs and other treatments. Patients with acute respiratory failure received oxygen therapy and, if necessary, ventilator support. Those who met the criteria for liver transplantation received a liver transplantation.

### Research strategy

Normal TSH level with an FT3 result below the lower limit of the reference interval (2.63 nmol/L) was defined as having low-T3 syndrome [23], and according to the dynamic changes in FT3 level, were divided into 3 types: (1) FT3 level is continuously higher than 2.63 pmol/L, FT3 level is persistently normal type; (2) FT3 level does not rise after decreasing (or the recovery amount is less than 15%), FT3 level is continuously decreasing type; (3) FT3 level is continuously decreasing type. The FT3 level was U-shaped and increased after the initial decrease. By comparing the proportions of different types of dynamic changes in the FT3 levels between the survival and the death groups, the effects of different types of FT3 changes on the 90 day survival of patients were compared using the patient survival curve. The receiver operating characteristic (ROC) curve was used to explore the value of the difference between a single change in FT3 level (single ΔFT3) and an extreme value change in FT3 level (ΔFT3 extreme difference) for predicting the 90 day prognosis of patients. Among these changes, for differences in FT3, TBil (mg/dL), INR, Cr (mg/dL) and Na, a single change is defined as the latter value minus the former value, which is represented by ‘△’.

### Statistical methods

Statistical analysis was performed using the SPSS 25.0 (IBM, Armonk, New York) and MedCalc 19 (MedCalc, Belgium) statistical analysis software. Continuous data were tested for normal distribution using the Kolmogorov–Smirnov test. Measurement data conforming to normal distribution were expressed as mean ± standard deviation (M ± SD), and the mean comparison between two groups was performed by t-test; non-normally distributed measurement data were expressed by the median and interquartile range (M, Q1, Q3) and the Mann–Whitney U test was used for analysis. Categorical data were expressed as frequencies (percentages) and analysed using the chi-square or Fisher’s exact tests. Survival analysis was used to compare the prognosis of different groups, a survival curve was drawn using the Kaplan–Meier method and a cumulative survival difference test was performed by log-rank test. Binary logistic regression univariate analysis was used to evaluate the influence degree of each factor, and the regression applied a stepwise forward method. The area under the ROC curve (AUROC) was used to compare the prognostic value of various models; *P* < 0.05 indicated a statistically significant difference.

## Results

### Patient baseline characteristics

A total of 122 patients with HBV-ACLF were included in this study and were divided into a survival and a death group according to their 90 day prognosis. The direct cause of death of the deceased patients was ‘ACLF’. There were 77 patients (63.1%) in the survival group, including 65 men (84.4%) and 12 women (15.6%), aged (43.66 ± 9.94) years; 45 (36.9%) patients were in the death group, including 35 men (77.8%) and 10 women (22.2%), aged (48.86 ± 8.91) years. The characteristics, primary laboratory results, organ failure and main prognostic scores of the two patient groups on admission are compared in Table 1. Age, liver cirrhosis ratio, AST, TBil, INR, Cr, Na, WBC, CTP, MELD, MELD-Na, CLIF-SOFA, CLIF-OF and AARC scores of the patients in the death group were significantly higher than those in the survival group (*P* < 0.05).

**Table 1:** Comparison of baseline levels between survival group and death group

	Survival group (77 cases)	Death group (45 cases)	Statistical value	P value
Clinical features				
Age (years)	43.66 ± 9.94	48.86 ± 8.91	t = 4.551	< 0.001
Gender (male/female)	65/12	35/10	Χ^2^ = 1.301	0.254
Chronic hepatitis B/liver cirrhosis	31/46	14/31	Χ^2^ = 6.702	0.010
Laboratory examination				
ALT(U/L)	151.64 ± 92.63	179.64 ± 94.89	t = 0.903	0.367
AST(U/L)	151.06 ± 86.85	220.12 ± 93.28	t = 2.016	0.045
TBil(mg/dL)	18.35 ± 9.01	28.31 ± 9.97	t = 9.375	< 0.001
INR	2.62 ± 1.49	3.94 ± 1.86	t = 4.902	< 0.001
Ln(HBV DNA)	3.82 ± 2.09	3.90 ± 1.52	t = 0.523	0.753
Cr(mg/dL)	0.72 ± 0.24	1.54 ± 0.48	t = 5.171	< 0.001
ALB(g/L)	32.34 ± 3.95	31.72 ± 4.19	t = 1.295	0.196
Na(mmol/L)	136.22 ± 3.67	133.65 ± 6.91	t = 4.514	< 0.001
WBC(× 10^9^/L)	7.05 ± 3.62	9.14 ± 4.87	t = 3.3698	0.001
Extrahepatic organ failure				
1 organ	25(32.5%)	21(46.7%)	Χ^2^ = 4.871	< 0.001
2 organs	6(7.8%)	10(22.2%)	Χ^2^ = 12.511	< 0.001
3 organs	1(1.3%)	6(13.3%)	Χ^2^ = 20.276	< 0.001
Prognosis score				
CTP	11.17 ± 1.27	12.27 ± 1.39	t = 7.074	< 0.001
MELD	26.48 ± 5.19	33.68 ± 4.87	t = 11.996	< 0.001
MELD-Na	27.18 ± 5.19	34.62 ± 4.24	t = 12.797	< 0.001
CLIF-SOFA	7.89 ± 1.78	10.97 ± 3.49	t = 10.830	< 0.001
CLIF-OF	9.19 ± 1.59	11.30 ± 2.07	t = 10.182	< 0.001
AARC	8.53 ± 1.78	11.18 ± 1.64	t = 12.824	< 0.001

P < 0.05 means the difference is statistically significant

### Differences in the types of dynamic FT3-level changes between the two groups

**Fig. 1:**
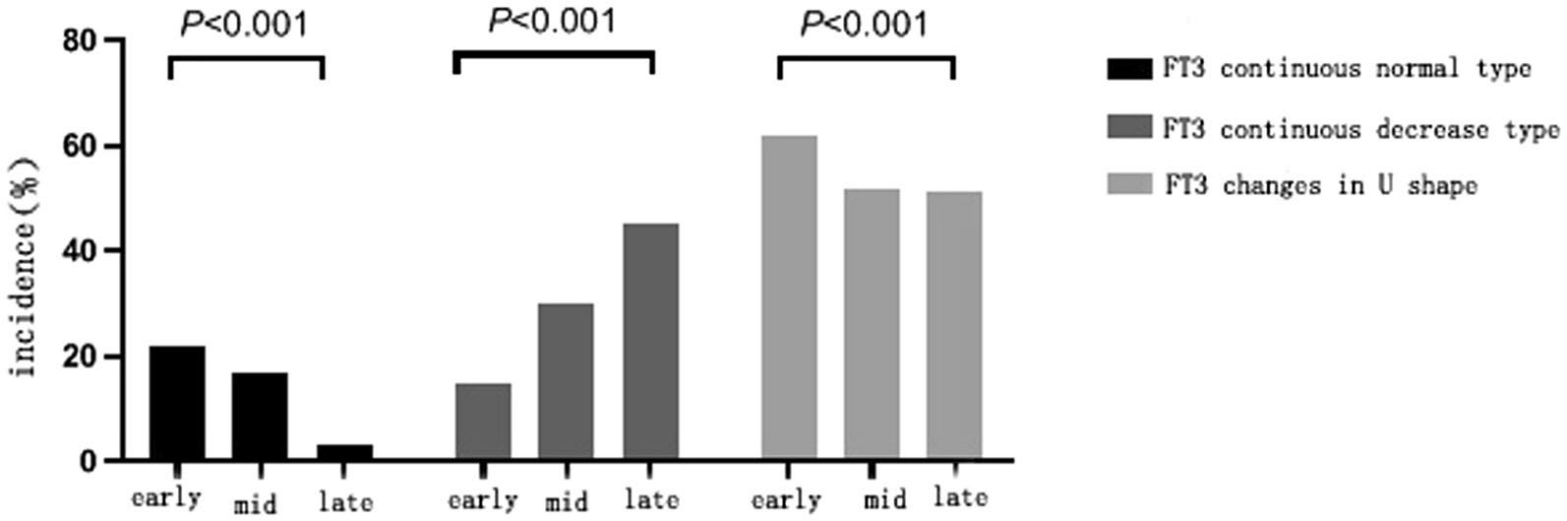
Comparison of the incidence of FT3 changes in patients with different stages of liver failure

Among the 122 patients included in the study, for 19 (15.6%) cases, the FT3 level continued to be normal, 35 (28.7%) cases had the continuous decrease type, and 68 (55.7%) cases reflected the U-shaped change; no other changes were found. The incidence of dynamic changes in FT3 levels in patients with different stages of liver failure is shown in Table 2 and Fig. 1. The results show that with the gradual increase of the liver failure stage, the incidence of persistent normal FT3 gradually decreased (*P* < 0.001), and the incidence of persistently decreased FT3 gradually increased (*P* < 0.001). The incidence of liver failure in patients with U-shaped changes in FT3 levels in the early stage was significantly higher than in the patients in the middle and late stages (*P* < 0.001). These results suggest that the type of FT3 dynamic changes may be associated with the severity of the disease. Table 3 shows the comparison results of the types of changes in FT3 levels in the survival and death groups. The incidence of persistent normal FT3-level changes in the survival group was significantly higher than in the death group (23.4% vs 2.2%, *P* < 0.001). The change in FT3-level was U-shaped and 62.3% in the survival group, significantly higher than the 24.4% recorded for the death group (*P* < 0.001). The changes in FT3 levels showed a continuous decrease and was 14.3% in the survival group, which was significantly lower than the 73.3% recorded for the death group (*P* < 0.001).

**Table 2:** Different stages of liver failure and types of FT3 level changes

	Stages of liver failure			*P* value
	Early stage (45 cases)	etaphase (46 cases)	Late stage (31 cases)	
FT3 continuous normal type (n, %)	10 (22.2%)	8 (17.4%)	1 (3.2%)	< 0.001
Continuous decrease of FT3 (n, %)	7 (15.6)	14 (30.4%)	14 (45.2%)	< 0.001
FT3 changes in U shape (n, %)	28 (62.2%)	24 (52.2%)	16 (51.6%)	< 0.001

P < 0.05 means the difference is statistically significant

**Table 3:** Comparison of FT3 level changes between survival group and death group

	Survival group (77 cases)	Death group (45 cases)	*P* value
FT3 continuous normal type (n, %)	18 (23.4%)	1 (2.2%)	< 0.001
FT3 continuous decrease type (n, %)	11 (14.3%)	33 (73.3%)	< 0.001
FT3 changes in U shape (n, %)	48 (62.3%)	11 (24.4%)	< 0.001

P < 0.05 means the difference is statistically significant

### The value of dynamic FT3-level changes in predicting the prognosis of patients

**Fig. 2:**
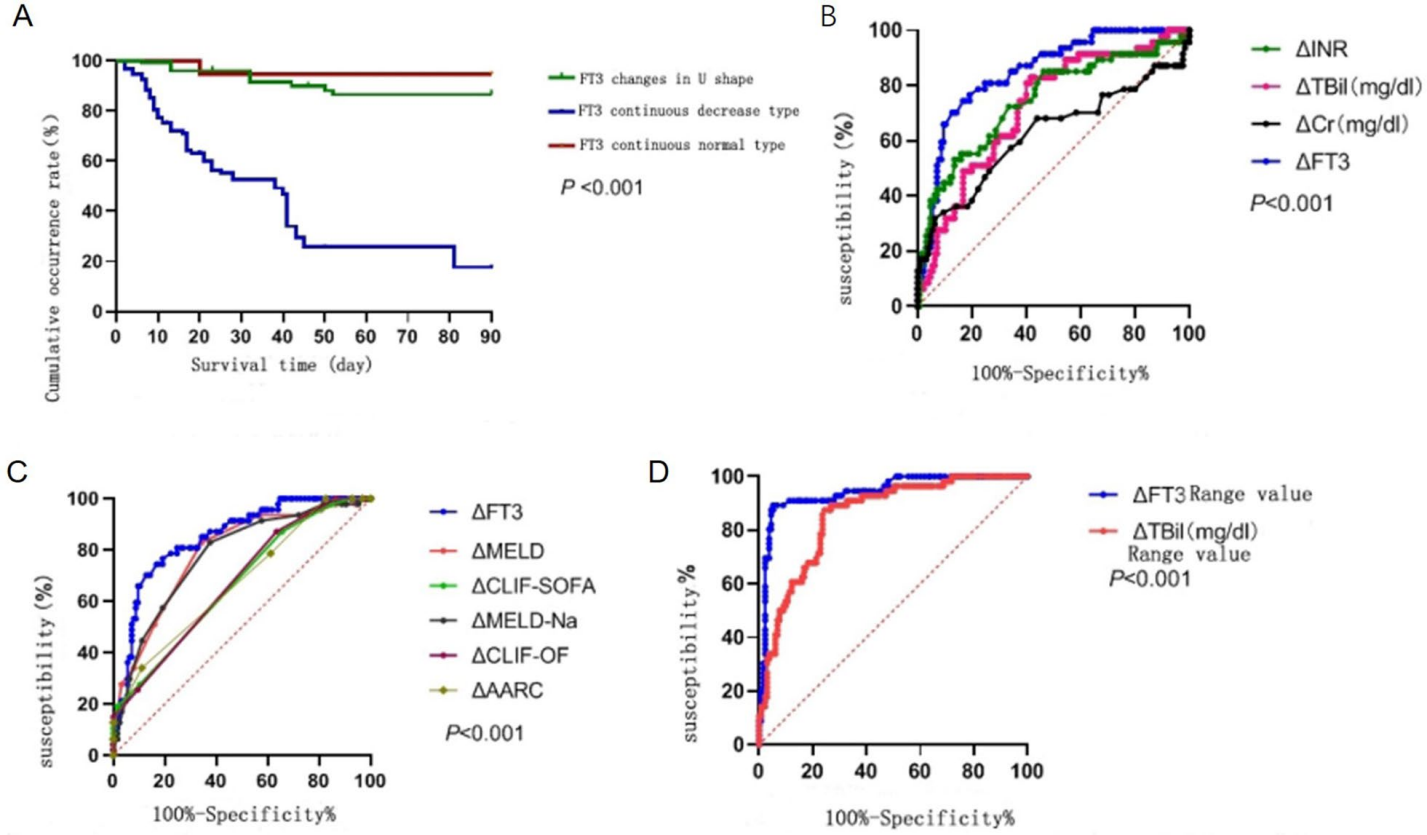
**A** Survival curves of patients with different FT3 changes. **B** Comparison of ROC curves of △FT3, △TBil, △INR, and △Cr on the prognosis of patients. **C** Comparison of ROC curves of △FT3 and single change of prognostic score on patient prognosis. **D** ROC curve of △FT3 range and △TBil range in predicting 90-day prognosis of patients

Kaplan–Meier survival curves showed significant differences between the survival curves of patients with three different types of dynamic changes in FT3 levels (*P* < 0.001). The 90 day prognostic risk of death in patients with persistently decreased dynamic FT3-level changes was significantly higher than in patients with persistently normal FT3 levels and a U-shaped curve (Fig. 2A), suggesting that the type of FT3 dynamic changes may be related to the 90 day prognosis of patients.

The MELD scoring system can more accurately subdivide the severity of liver cirrhosis and can more precisely determine the prognosis of patients with end-stage liver disease [24]. In this study, FT3 levels were detected and MELD scores were calculated every 4 days. The difference between the final MELD score and the highest/lowest MELD score was used to obtain the type of MELD score change as follows: MELD score decreased type (73 cases); MELD score increased type (49 cases). Compared with the group with an elevated MELD score (77.6%), the incidence of persistently decreased FT3-level changes in the group with decreased MELD scores (8.2%) was significantly lower (*P* < 0.001). This further suggests that the type of dynamic changes in FT3 levels may be associated with changes in patients' conditions (Table 4).

**Table 4:** Relationship between MELD score change type and FT3 change type

	MELD score decreased (73 cases)	MELD score increased (49 cases)	P value
FT3 continuous normal type (n, %)	18 (24.7%)	1 (2.0%)	< 0.001
FT3 continuous decrease type (n, %)	6 (8.2%)	38 (77.6%)	< 0.001
FT3 changes in U shape (n, %)	49 (67.1%)	10 (20.4%)	< 0.001

P < 0.05, the difference is considered statistically significant

### The value of a single △FT3 in predicting the 90 day patient prognosis

Binary logistic regression univariate analysis showed that a single instance of ΔFT3 was an independent protective factor affecting the 90 day prognosis of patients with HBV-ACLF (odds ratio [OR] value 0.033 (0.010–0.110), *P* < 0.001), while a single instance of ΔTBil (mg/ dL) (*P* < 0.001), single △INR (*P* < 0.001) and a single △Cr (mg/dL) (*P* < 0.001) were independent risk factors affecting the 90 day prognosis of patients with HBV-ACLF (Table 5).

**Table 5:** Binary logistic regression analysis of single change affecting patients’ 90 day prognosis

	Coefficient	Wald value	OR (95% CI)	P value
Single △FT3(umol/L)	− 3.39	31.29	0.033 (0.010–0.110)	< 0.001
Single △TBil(mg/dL)	0.138	14.973	1.148 (1.071–1.232)	< 0.001
Single △INR	0.137	6.070	1.147 (1.028–1.279)	0.014
Single△Cr(mg/dL)	1.068	4.680	2.910 (1.106–4.659)	0.031
Single△Na(mmol/L)	− 0.031	0.530	0.969 (0.891–1.054)	0.467

P < 0.05, the difference is considered statistically significant

After drawing the ROC curve, the AUROC of a single △FT3 was derived as 0.848 (95% confidence interval [CI] 0.786–0.910; *P* < 0.0001); the AUROC of a single △TBil (mg/dL) was 0.720 (95% CI 0.636–0.864; *P* < 0.0001); the AUROC of a single △INR was 0.745 (95% CI 0.657–0.833; *P* < 0.0001); and the AUROC of a single △Cr (mg/dL) was 0.624 (95% CI 0.518–0.730) (*P* = 0.013) (see Fig. 2B). The results show that a single instance of ΔFT3, ΔTBil (mg/dL), ΔINR, ΔCr (mg/dL) and ΔNa could predict the 90 day prognosis of patients with HBV-ACLF, but a single dose of the prediction effect of △FT3 on the 90 day prognosis of patients was better than a single △TBil (mg/dL), △INR and △Cr (mg/dL). Similarly, a single ΔFT3 was better than single ΔMELD, ΔMELD-Na, ΔCLIF-SOFA, ΔCLIF-OF and ΔAARC scores in predicting the patient’s 90 day prognosis (*P* < 0.0001) (Fig. 2C).

### The value of the dynamic change range in FT3 level (△FT3 range) for predicting patients’ 90 day prognosis

Binary logistic regression univariate analysis was used to explore the prediction of the △FT3, △TBil (mg/dL), △INR, △Cr (mg/dL) and △Na ranges and their 90 day prognostic impact on patients with HBV-ACLF (see Table 6).

**Table 6:** Binary logistic single factor regression analysis of extreme value changes affecting patients’ 90 day prognosis

	Coefficient	Wald value	OR(95%CL)	P value
△ FT3 extreme value	− 5.127	29.69	0.006 (0.001–0.023)	< 0.001
△ TBIL extreme value	0.196	11.46	1.216 (1.086–1.362)	0.001
△ INR range	0.060	0.711	1.062 (0.923–1.222)	0.399
△ CR extreme difference	− 0.095	0.038	0.909 (0.346–2.386)	0.846
△ Na range	− 0.001	0.001	0.999 (0.877–1.138)	0.986

P < 0.05, the difference is considered statistically significant

The results show that the OR value of the △FT3 range was 0.006 (0.001–0.023) (*P* < 0.001), and the OR value of the △TBil (mg/dL) range was 1.216 (1.086–1.362) *(P* < 0.001). This suggests that the ΔFT3 range is an independent protective factor affecting the 90 day prognosis of patients with HBV-ACLF, and the ΔTBil (mg/dL) range is an independent risk factor.

**Fig. 3:**
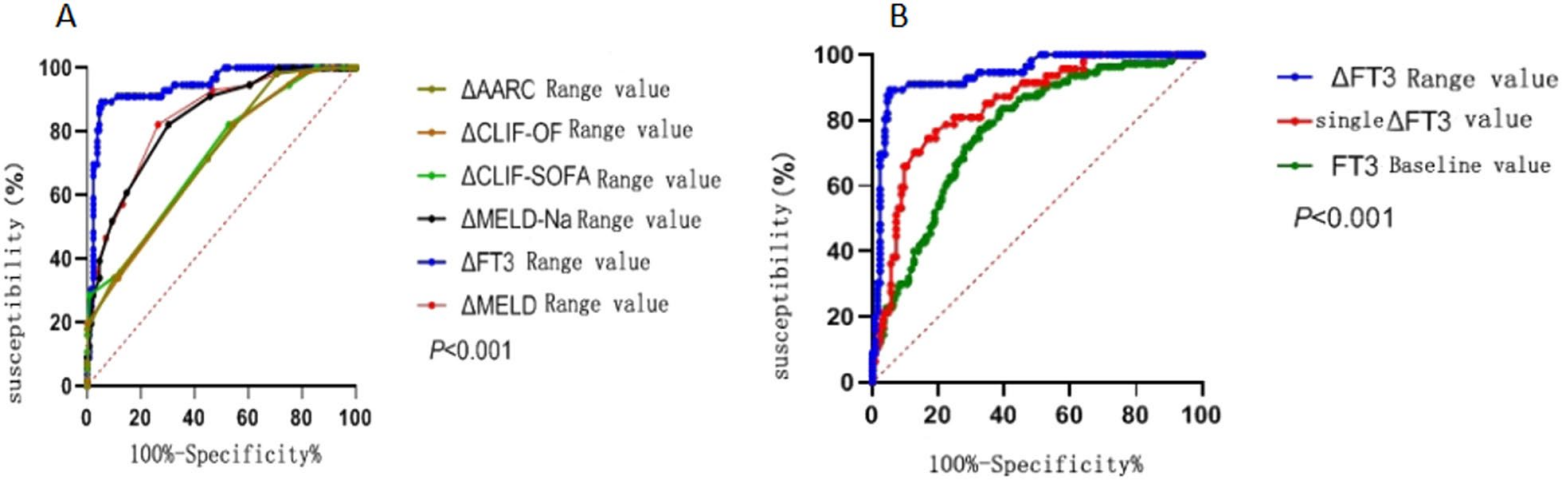
**A** ROC curve of △FT3 range and prognostic score range for 90 day prognosis evaluation of patients. **B** ROC curve of FT3 baseline value, single △FT3 value and △FT3 extreme difference value predicting the prognosis of patients at 90 days

The ROC curve results show that the AUROC of the △FT3 range was 0.942 (95% CI 0.906–0.980) (*P* < 0.0001), and the AUROC of the △TBil (mg/dL) range was 0.854 (95% CI 0.780–0.910) (P < 0.0001) (see Fig. 2D). This suggests that the ΔFT3 is better than the ΔTBil (mg/dL) range for predicting the 90 day prognosis of patients. Compared with the predictive value of the △MELD score range, the △MELD-Na, △CLIF-SOFA, △CLIF-OF and △AARC score ranges, the △FT3 range has poor predictive value. △MELD score range, the △MELD-Na, △CLIF-SOFA, △CLIF-OF and △AARC score ranges were better for predicting the 90 day prognosis of patients (*P* < 0.0001) (Fig. 3A).

### Comparison of the value of the baseline FT3 level, single △FT3 value and △FT3 extreme difference for predicting the prognosis of patients at 90 days

The ROC curve of the baseline FT3 level, the single ΔFT3 and the ΔFT3 extreme values for predicting the prognosis of patients at 90 days were drawn (see Fig. 3B). The results show that the AUROC of the baseline FT3 level value was 0.780, (95% CI 0.731–0.829; *P* < 0.001), the Youden index was 0.461 (95% CI 0.343–0.529), the sensitivity was 86.1% and the specificity was 59.9%. The AUROC of a single △FT3 was 0.848 (95% CI 0.786–0.910) (*P* < 0.0001), the Youden index was 0.577, the sensitivity was 74.5% and the specificity was 83.2%. The AUROC of the △FT3 range was 0.942 (95% CI 0.906–0.980; *P* < 0.0001), the Youden index was 0.840, the sensitivity was 89.3% and the specificity was 94.7%. These results suggest that the ΔFT3 extreme value is more effective for predicting the prognosis of patients at 90 days than the baseline FT3 level and the single ΔFT3 values.

## Discussion

Hepatitis B virus associated ACLF is a complex syndrome with extremely high short-term mortality, characterised by acute deterioration of liver functioning and eventual progression to multiorgan failure [25]. To reduce HBV-ACLF mortality, patients with a poor prognosis must be accurately identified for early treatment [6]. In the current research on the prognosis of ACLF patients, most predictive models are calculated based on the baseline detection values of patients on admission [10]. However, ACLF has a long disease course. During treatment after admission, in addition to first-strike factor management, the prevention and treatment of the patient's complications, liver regeneration and other factors also affect their outcome. Therefore, theoretically, the use of dynamic data can more accurately reflect changes in the patient's condition and predict their prognosis.

Thyroid hormone receptor 3 and FT3 play crucial roles in metabolic homeostasis [26]. A cohort study found that patients with thyroid disease with suspected NAFLD had higher FT3 levels, lower FT4 levels and a higher FT3/FT4 ratio [27], suggesting the predictive value of FT3 in liver disease prognosis. Other studies found that low T3 syndrome, caused by decreased FT3, is associated with high mortality rates that involve, for example, acute myocardial infarction [28], decompensated cirrhosis and ACLF [29]. This study explored the predictive value of dynamic changes in FT3 levels on the 90 day prognosis of patients with HBV-ACLF. The results found only three types of dynamic changes in FT3 levels in all of the participants: FT3 levels continued to be normal, FT3 levels continued to decrease and FT3 levels showed U-shaped changes. No other types of FT3-level changes were observed. With the gradual progression of liver failure stages, the incidence of persistent normal FT3 gradually decreased (*P* < 0.001), and the incidence of persistently decreased FT3 gradually increased (*P* < 0.001). The incidence of FT3 with U-shaped changes was significantly higher in the early stage of liver failure compared with in the middle and late stages. It is suggested that the type of FT3 dynamic change may be related to the severity of the patient's disease. From the 90 day prognosis analysis of patients, the survival curves of the two groups of patients with FT3 changes were significantly different (*P* < 0.001). The dynamic FT3 changes may be an important factor affecting the prognosis of patients; specifically, the disease of patients indicating U-shaped changes may more likely be reversible. At present, the exact mechanism of the relationship between FT3 level and the severity and prognosis of patients with HBV-ACLF remains unclear. According to the literature reports, another feature of ACLF is that it is potentially reversible, and patients can recover from acute shock to the basic state before the disease progresses [30]. Recovery to a pre-disease state and ultimately no need for liver transplantation is thus possible. In cases of patients with severe hepatitis, the abnormal changes in serum thyroid hormone levels will be more obvious; once the disease improves, thyroid hormone levels will gradually move towards normal again [31].

Considering the factors that affect patient prognosis, among the commonly used indicators and comprehensive scores for evaluating severity in patients with liver failure, TBil, INR, Cr (mg/dL) and Na are all recognised as important parameters. Research by Yang [32] and other studies have shown that dynamic changes in TBil and PT in patients with HBV-ACLF within 8 days after admission are helpful for the early prognosis of patients. Consistent with previous studies, the results of this study showed that a single dose of ΔTBil (mg/dL), ΔINR and ΔCr (mg/dL) were independent risk factors for the 90 day prognosis of patients with HBV-ACLF. This study also found that although a single dose of ΔFT3, ΔTBil (mg/dL), ΔINR, ΔCr (mg/dL) and ΔNa could predict the 90 day prognosis of patients with HBV-ACLF, the evaluation effect of a single △FT3 for predicting prognosis was better than that of a single △TBil (mg/dL), △INR and △Cr (mg/dL). For dynamic changes in FT3 levels, we found that the ΔFT3 range was also an independent protective factor affecting the 90 day prognosis of patients with HBV-ACLF. Compared with the ΔTBil (mg/dL) range for predicting patients’ 90 day prognosis, the ΔFT3 range provided a better evaluation value. Dynamic change in the FT3 level showed greater predictive value for the 90 day prognosis of patients with HBV-ACLF compared with other indicators.

Various scoring systems have been developed to evaluate the prognosis of patients with HBV-ACLF. However, these may impose limitations on the prognosis of HBV-ACLF [33]. For example, in the evaluation parameters of CTP score, ascites and HE are subjective variables, the degree of ascites and serum ALB level are easily affected by treatment and their score ranges are narrow and have limited resolving power [34, 35]. The CLIF-SOFA and CLIF-COF are scoring models based on the European population, patients with alcoholic liver disease and hepatitis C; there is also a degree of ethnic and aetiology heterogeneity involved, questioning their broad application [36]. The AARC score is based on Asian populations and does not consider circulatory and renal factors, nor the role of inflammatory responses in the development of ACLF; accordingly, its accuracy has been criticised [37]. The MELD score and MELD-Na score have the advantages of objective parameters and are often used for deriving a prognosis for ACLF patients [6]; however, they do not consider HE, hepatorenal syndrome, the influence of ascites or upper gastrointestinal bleeding on prognosis, and the influence of treatment methods, such as diuretics and artificial livers are not considered, rendering their accuracy for evaluating the prognosis of patients with ACLF questionable [12]. A study conducted by Zhang Li et al. [38] showed that the MELD score 5–7 days after a diagnosis of ACLF was higher than the baseline level when predicting the 90 day prognostic value of patients with HBV-ACLF. Research conducted by Wang et al. [39] showed that the MELD score difference after 1 week following admission could better reflect the severity of the disease than the MELD score baseline value. Dynamic observation of MELD score difference has important significance for predicting a survival time of 8, 12 and 24 weeks in patients with HBV-related liver failure. This study showed that the AUROC of the single △MELD score and the single △MELD-Na score were all greater than 0.7, indicating good predictive value. This is consistent with the results of previous studies [39]. This study also found that the value of a single ΔFT3 on the 90 day prognosis of patients was better than single △MELD, △MELD-Na, △CLIF-SOFA, △CLIF-OF and △AARC scores, and the △FT3 range was better than △MELD, △MELD-Na, △CLIF-SOFA and △CLIF-OF score ranges for evaluating patients’ 90 day prognosis. These findings suggest that dynamic changes in FT3 levels have important predictive value for the 90 day prognosis of patients with HBV-ACLF. In addition, by comparing the effect of the FT3 baseline, single ΔFT3 and ΔFT3 extreme values for predicting patients’ 90 day prognosis, it was found that the effect of ΔFT3 range value was better than the baseline value of FT3 level and the single ΔFT3 value. However, there are few reports on the effect of ACLF extreme value or the extreme value of the current commonly used prognostic score model on the 90 day prognosis of patients, which may indicate a new direction for exploring the short and long-term prognostic factors of patients with HBV-ACLF.

This study has some limitations. The sample size was small; future research should increase the sample size to further verify our conclusions. Second, our study was a single-centre case-control study, which has limitations concerning the generalisation and applicability of the conclusions and should be further explored using a multi-centre prospective cohort study or a randomised control study after FT3 interventional treatment. Finally, future research should incorporate more scoring systems, including CLIF, APACHE and NACSELD scores, which is expected to be a multivariate analysis was performed to account for the many well-documented variables in the future. This study is limited to hypothesis-generating data, for which larger retrospective studies and/or prospective observational studies may be needed to further elucidate the potential role of FT3 in risk stratification and/or its use in clinical decision-making for patients with HBV-ACLF.

## Conclusion

The types of dynamic FT3 changes are associated with disease severity and 90 day prognosis in patients with HBV-ACLF. Both the single ΔFT3 value and the ΔFT3 range value had a good effect on predicting the 90 day prognosis of patients with HBV-ACLF. Accordingly, the study results reflect clinical application prospects.

## What is already known on this topic?

Establishing an effective analytical model for predicting the prognosis of patients with HBV-ACLF, which can truly, objectively and accurately reflect the patient's condition. Additionally, identifying differences in prognosis is a key issue in the process of HBV-ACLF treatment. Free triiodothyronine (FT3), as one of the humoral regulating hormones, plays an important role in regulating the homeostasis of patients. Studies have shown that the use of dynamic data can more accurately reflect changes in patients' conditions and predict their prognosis.

## What this study adds

There is insufficient evidence that dynamic changes in FT3 levels can be used to predict the prognosis of patients with HBV-ACLF and few related studies have been conducted. This study found that the single ΔFT3 value and the ΔFT3 range value had a good effect on predicting the 90 day prognosis of patients with HBV-ACLF.

## How this study might affect research, practice or policy?

This study selected hospitalised patients with HBV-ACLF to conduct an observational case-control study exploring the type of dynamic changes in FT3 levels and the difference between dynamic changes for predicting the 90 day prognosis of patients with HBV-ACLF and provide data support for doing so.

## Data Availability

All data generated or analyzed during this study are included in this article.
